# Neuro-Cognitive Differences in Semantic Processing Between Native Speakers and Proficient Learners of Mandarin Chinese

**DOI:** 10.3389/fpsyg.2021.781304

**Published:** 2021-11-18

**Authors:** Chia-Ho Lai, Shu-Kai Hsieh, Chia-Lin Lee, Lily I-Wen Su, Te-Hsin Liu, Chia-Rung Lu, I-Ni Tsai, Tai-Li Chou

**Affiliations:** ^1^Department of Psychology, New York University, New York, NY, United States; ^2^Department of Psychology, National Taiwan University, Taipei, Taiwan; ^3^Graduate Institute of Linguistics, National Taiwan University, Taipei, Taiwan; ^4^Graduate Program of Teaching Chinese as a Second Language, National Taiwan University, Taipei, Taiwan; ^5^Neurobiology and Cognitive Science Center, National Taiwan University, Taipei, Taiwan

**Keywords:** learning, semantic, proficiency, brain, connectivity

## Abstract

The present study aimed to investigate the neural mechanism underlying semantic processing in Mandarin Chinese adult learners, focusing on the learners who were Indo-European language speakers with advanced levels of proficiency in Mandarin Chinese. We used functional magnetic resonance imaging technique and a semantic judgment task to test 24 Mandarin Chinese adult learners (L2 group) and 26 Mandarin Chinese adult native speakers (L1 group) as a control group. In the task, participants were asked to indicate whether two-character pairs were related in meaning. Compared to the L1 group, the L2 group had greater activation in the bilateral occipital regions, including the fusiform gyrus and middle occipital gyrus, as well as the right superior parietal lobule. On the other hand, less activation in the bilateral temporal regions was found in the L2 group relative to the L1 group. Correlation analysis further revealed that, within the L2 group, increased activation in the left middle temporal gyrus/superior temporal gyrus (M/STG, BA 21) was correlated with higher accuracy in the semantic judgment task as well as better scores in the two vocabulary tests, the Assessment of Chinese character list for grade 3 to grade 9 (A39) and the Peabody Picture Vocabulary Test-Revised. In addition, functional connectivity analysis showed that connectivity strength between the left fusiform gyrus and left ventral inferior frontal gyrus (IFG, BA 47) was modulated by the accuracy in the semantic judgment task in the L1 group. By contrast, this modulation effect was weaker in the L2 group. Taken together, our study suggests that Mandarin Chinese adult learners rely on greater recruitment of the bilateral occipital regions to process orthographic information to access the meaning of Chinese characters. Also, our correlation results provide convergent evidence that the left M/STG (BA 21) plays a crucial role in the storage of semantic knowledge for readers to access to conceptual information. Moreover, the connectivity results indicate that the left ventral pathway (left fusiform gyrus-left ventral IFG) is associated with orthographic-semantic processing in Mandarin Chinese. However, this semantic-related ventral pathway might require more time and language experience to be developed, especially for the late adult learners of Mandarin Chinese.

## Introduction

Around the world, many people could speak multiple languages other than their mother tongue. Though an early view on language acquisition suggested that there is a critical period for learning a language (Lenneberg, 1967), many people are able to acquire another language in their late adulthood and even achieve native-like proficiency. The corresponding neuronal changes induced by the experience of second language acquisition have been one of the crucial topics in the study of bilingualism (Li et al., 2014). The present study aimed to investigate the neural correlates in the proficient-level Mandarin Chinese adult learners, who speak alphabetic languages as their native languages, during understanding words.

Compared to the Indo-European languages, Mandarin Chinese is unique for its logographic system (Wu and Chou, 2000; Reich et al., 2003). Chinese characters are monosyllabic and consist of smaller units like strokes and radicals with certain spatial arrangements. In addition, Mandarin Chinese is a tonal language with four lexical tones. Speakers of Mandarin Chinese rely on these lexical tones to differentiate characters or phrases during a conversation. Neuroimaging studies have shown similarities and dissimilarities between Mandarin Chinese and other alphabetic languages, such as English. For example, some common brain regions have been found in both English and Mandarin Chinese during semantic processing, including the left inferior frontal gyrus (IFG, BA45/47), left posterior middle temporal gyrus (MTG, BA21), and left inferior parietal lobule (BA39/40; Chou et al., 2006a,b, 2009). By contrast, some studies have pointed out that there are brain regions specific to the processing of Mandarin Chinese. For instance, compared to English reading, more activation in the right middle occipital gyrus (MOG) and right fusiform gyrus was found during Chinese reading (Bolger et al., 2005; Mei et al., 2015). Nelson et al. (2009) found that, for English native speakers who learned Mandarin Chinese for 1year, the left fusiform areas were activated when they read English stimuli, whereas the bilateral fusiform areas were activated when they read Chinese stimuli. These studies suggested that when viewing Mandarin Chinese, readers tend to recruit more brain regions associated with visuospatial analysis for processing orthographical information of Chinese characters.

Recently, several studies have investigated the neuronal changes of learning Mandarin Chinese by short-term language training (Wang et al., 2003; Wong et al., 2007; Deng et al., 2008, 2011; Qi et al., 2015; Yang et al., 2015). Wang et al. (2003) conducted Mandarin Chinese tone training in six native English speakers. Participants were asked to perform the tone identification task during the functional magnetic resonance imaging (fMRI) scanning sessions before and after training. They found that participants’ performance improvements in the tone identification task were related to the increased activation in the left superior temporal gyrus (STG, BA22) and right IFG (BA44). Wong et al. (2007) also found increased activation in the left STG in the successful learners as compared to the less successful learners in a word-learning paradigm, in which participants were trained to discriminate the meanings of artificial words by tonal differences. These studies suggested that the left STG might play a role in identifying lexical tones. In addition to tone learning, Deng et al. (2008) examined the Chinese characters learning in English native speakers. During their training, participants were asked to learn six lists of Chinese characters with their translational meanings in English. Their fMRI result showed that participants elicited greater activation in the left fusiform gyrus, left IFG, and left superior parietal lobule (SPL) after training. They suggested that the activated left fusiform gyrus was responsible for orthographic processing in Mandarin Chinese, whereas the left IFG was responsible for the retrieval and manipulation of semantic representations. Also, the left SPL might be associated with learning the visual-spatial relations of Chinese characters and transferring the knowledge of semantic radicals to the novel characters. These studies provided some neural evidence for short-term training effects in learning Mandarin Chinese as a second language; however, the long-term effects of second language learning and related changes in the brain are less understood in the current literature.

The primary research question of the present study is to examine the influence of learning Chinese as a second language on the ventral pathway with semantic processing. A prominent feature of Chinese words is the mapping from orthography to semantics (Cao et al., 2017; Chou and Booth, 2020). The relation between form and meaning in English admits many exceptions and lacks the reliability of semantic information at the sublexical level. In contrast, Chinese includes greater semantic information, such as semantic radicals at the sublexical level, showing a more direct mapping between orthography and semantics. Thus, it is crucial to observe the effect of learning Chinese as a second language on the neural substrates of semantic processing, particularly the ventral pathway (Wang et al., 2019). The ventral pathway has been associated with semantic processing, including the middle occipital gyrus, fusiform gyrus, middle/superior temporal gyrus, and IFG (Fan et al., 2020; Nichols et al., 2021).

Moreover, the second research question is to examine semantic processing in the ventral pathway for Mandarin Chinese adult learners with advanced proficiency levels. As most imaging studies of semantic processing in learning Chinese focus on short-term influences (review in Chung et al., 2019), the present study chooses to examine long-term effects on the ventral pathway (i.e., longer than 5years). The chosen learning length of 5years is based on previous imaging studies of semantic development in Chinese using cross-sectional (Lee et al., 2015; Chen et al., 2016; Fan et al., 2020) and longitudinal approaches (Lee et al., 2016; Chou et al., 2019; Fan et al., 2021). The long-term learning places substantial impacts on the ventral pathway. For example, a developmental increase in brain activation has been found in the left IFG and middle/superior temporal gyrus (Wong et al., 2019). In addition, dynamic interaction between brain regions, such as functional connectivity, shows developmental increases on the ventral pathway during semantic judgments (Fan et al., 2021). Taken from developmental implications, it is thus important to examine long-term learning effects on the ventral pathway with semantic processing.

The goal of the present study was to investigate the neural mechanisms of semantic processing in Mandarin Chinese adult learners with advanced proficiency levels. In particular, we focused on the ventral pathway to examine the mapping from orthography to semantics in Chinese (Cao et al., 2017; Chou and Booth, 2020). Also, we recruited late adult learners whose native languages were alphabetic languages to observe long-term effects on the ventral pathway. The regions of interest on the ventral pathway were the left IFG, middle/superior frontal gyrus (SFG), fusiform gyrus, and middle occipital gyrus. We expected to see the group differences in brain activity and functional connectivity along the ventral pathway between the L1 and L2 groups (Fan et al., 2021). Furthermore, we were interested in examining the individual differences among late adult language learners (Johnson and Newport, 1989; Gao et al., 2017; Chung et al., 2019). Two vocabulary tests were used to assess the individual’s semantic knowledge and examined the associated neural correlates of proficiency in Mandarin Chinese adult learners. We expected to see the individual differences in brain activity and functional connectivity along the ventral pathway in the L2 group.

## Materials and Methods

### Participants

Twenty-six Mandarin Chinese adult native speakers (L1 group, 10 females, age=24.1±3.1years old, age range=20–31years old) and 24 Mandarin Chinese adult learners (L2 group, 4 females, age=25.0±3.7years old, age range=20–32years old) participated in the study. The L2 participants were all native speakers of Indo-European languages.^1^ Based on their self-report, the L2 participants started learning Mandarin Chinese when they were in high school or college (average age of acquisition: 19.2±3.7years old, age range: 14–29years old). The informed consent procedures were approved by the Research Ethics Committee at the National Taiwan University.

### Language Proficiency Tests

To assess L2 participants’ proficiency in reading comprehension, we adapted the reading section of the intermediate-to-advanced-level mock test from the Test Of Chinese as a Foreign Language (TOCFL). TOCFL is a standardized test developed by the National Chinese Test Promotion Working Committee.^2^ The average score of the reading test was 93.7±7.5 [score range: 76–100 (total score: 100)], indicating that all the participants in the L2 group had the intermediate-advanced level of reading proficiency in Mandarin Chinese.

The Assessment of Chinese character list for grade 3 to grade 9 (A39) was used to evaluate L2 participants’ expressive vocabulary in Mandarin Chinese. The A39 is developed to estimating the vocabulary size of Chinese characters from grade 3 to grade 9 (Hung et al., 2008). There are 40 items of Chinese characters in the assessment list. For each item, participants were asked to name the character and use it as a morpheme to produce a disyllabic or trisyllabic word. The reliabilities of A39 (estimated by test-retest, split-half, coefficient alpha) were all over 0.85 in each grade. The L2 participants’ average score of A39 was 20.0±7.4 (score range: 11.5–38.5).

In addition, the Chinese version of Peabody Picture Vocabulary Test-Revised (PPVT-R) was used to evaluate L2 participants’ receptive vocabulary (Sun et al., 2021). There are 125 items in the test. In each trial, participants were asked to listen to a word uttered by the experimenter and then chose from one of the four pictures that matched the word’s meaning. The test-retest reliability of PPVT-R was 0.84 (Lu and Liu, 1998). The L2 participants’ average score of PPVT-R was 81.3±18.5 (score range: 32–109). Participants completed all the tests before the MRI session.

### Functional Task

The function task used an event-related design for stimuli presentation. Participants performed a semantic judgment task in the MRI scanner. The task included semantically related pairs and semantically unrelated pairs (Fan et al., 2010). Forty-eight character pairs were semantically related according to their free association values (mean=0.14, SD=0.13, ranging from 0.73 to 0.01; Hue et al., 2005). Twenty-four character pairs were semantically unrelated with zero association values. In the task, participants were asked to indicate whether the two Chinese characters are related in meaning. Each trial started with a solid square for 500ms, followed by the first character (800ms), a blank interval (200ms), and the second character (3,000ms). Participants were instructed to make a response during the presentation of the second character. In addition, we included 24 Chinese pairs as the perceptual control condition. In this condition, two-word Chinese pairs were presented sequentially with the same trial procedure, and participants were asked to indicate whether the pair of stimuli were identical or not. The yes/no responses were counterbalanced across conditions. For the following fMRI analyses, we compared the semantic-related pairs with perceptual control pairs as a baseline to control for the orthographic information of Chinese characters. Then, we used this contrast to examine semantic processing in both L1 and L2 groups and their group differences (Lee et al., 2011).

### MRI Data Acquisition

Participants lay in the scanner with their head positions secured. The head coil was positioned over their heads. The optical response box was placed on their right hands. The visual stimuli were presented to participants by the projection goggles. MRI data were acquired using a 3-Tesla Prisma Siemens scanner with a 20-channel head coil at Imaging Center for Integrated Body, Mind and Culture Research in National Taiwan University. Gradient-echo localizer images were acquired to determine the placement of the functional slices. For the functional imaging studies, a susceptibility weighted single-shot EPI (echo planar imaging) method with BOLD (blood oxygenation level-dependent) was used. Functional images were collected parallel to AC-PC plane with interleaved whole-brain EPI acquisition from bottom to top. The following scan parameters for functional images were used: TR=2000ms, TE=24ms, flip angle=90°, matrix size=64×64, field of view=192, slice thickness=3mm, and number of slices=36. Each participant performed two functional runs, and each run had 134 image volumes (4.47min/run, total: 8.9min). A high-resolution, T1-weighted 3D image was also acquired using following parameters: TR=2000ms, TE=2.3ms, flip angle=8°, matrix size=256×256, field of view=240, slice thickness=0.94mm, and number of slices=192.

### Image Data Analysis

Data analysis was performed using Statistical Parametric Mapping software (SPM8). In data preprocessing, the functional images were corrected for differences in slice acquisition time to the middle volume and were realigned to the first volume in the scanning session using affine transformations. No participant had more than 3mm of movement in any plane. The co-registered images were normalized to the MNI (Montreal Neurological Institute) average template. Then, the normalized functional images were smoothed with a 10mm full width at half maximum of the Gaussian kernel. Statistical analyses were calculated on the smoothed data with a high pass filter (128-s cutoff period) in order to remove low-frequency artifacts. For whole-brain analysis, data from each participant were entered into a general linear model using an event-related analysis procedure. Character pairs were treated as individual events and were convolved with a canonical hemodynamic response function in the model. There were three event types: semantic-related, semantic-unrelated, and perceptual control. The contrast between semantic-related and perceptual control was created for each individual participant. Considering the statistical power should be equal between conditions, the incorrect trials were included for the analyses (Bitan et al., 2007; Lee et al., 2011; Chen et al., 2016). The resulting model coefficients from the contrast of individual subjects were entered into subsequent second-order random effects analyses. We then used one-sample and two-sample t tests to examine semantic processing within each group (L1 group and L2 group) and between groups, respectively. We conducted a Monte Carlo simulation with 10,000 iterations to determine the threshold for multiple comparison correction (Slotnick et al., 2003). The reported areas in the whole-brain analysis were set to a voxel-wise threshold of *p*=0.005 with a cluster extent of 100 voxels to achieve the FWE-corrected threshold of *p*<0.05 at the cluster level.

### Correlation Analysis

To further investigate the relationship between the semantic processing and the individual difference of proficiency, we extracted the beta values of the highest peak in the *a priori* region of interest (ROI) in the ventral pathway from the group comparison results. The ROIs include the left IFG, left middle temporal gyrus/superior temporal gyrus (M/STG), left MOG, and left fusiform gyrus. These beta values were correlated with the L2 participants’ vocabulary scores as well as their accuracy and reaction time in the semantic judgment task.

### ROI-Based Connectivity Analysis

Previous studies have shown the functional connectivity between the left fusiform gyrus and left ventral IFG (BA 47) during lexical/semantic-related tasks in Mandarin Chinese native speakers (Wang et al., 2019; Fan et al., 2020). To test whether the proficient Mandarin Chinese learners would show similar connectivity in the ventral language pathway, we conducted a generalized psychophysiological interaction (gPPI) analysis (McLaren et al., 2012) with the left fusiform gyrus as the seed region. We used a sphere of 6mm radius centered on the coordinate of the left fusiform gyrus [−42–49−15] from the group comparison results in the current study. For the gPPI analysis, the deconvolved time series were extracted from the seed region and were entered in the GLM model as the physiological variable. The three task conditions were entered as the psychological variables. The product of the time series signals and the conditions were entered as the interaction terms in the model. We then specified the contrast of semantic-related interaction term versus perceptual control interaction term at the individual level. Based on our *a priori* hypothesis, a sphere of 6mm radius centered on the left ventral IFG (BA 47) [−42, 33–1] from a meta-analysis of neuroimaging studies on Mandarin Chinese (Wu et al., 2012) was used as the target region. We extracted the individual participants’ connectivity strengths between the seed region and the target region for further analyses.

## Results

### Behavioral Results

In the L1 group, the accuracies (mean±*SD*) for the related, unrelated, and perceptual conditions were 96±6%, 97±5%, and 99±3%. In the L2 group, the accuracies (mean±*SD*) for the related, unrelated, and perceptual conditions were 69±12%, 84±12%, and 97±6%. A 2 group (L1, L2)×3 condition (related, unrelated, and perceptual) ANOVA on accuracy was performed. The results showed a main effect of group [*F*(1, 48)=98.86, *p*<0.001], a main effect of condition [*F*(2, 96)=47.03, *p*<0.001], and an interaction between group and condition [*F*(2,96)=31.41, *p*<0.001]. The further simple main effect analyses showed that both L1 and L2 groups had similar accuracy in the perceptual condition [*F*(1, 144)=0.93, *p*<0.337]. However, compared to the L1 group, the L2 group were less accurate in the related [*F*(1, 144)=136.40, *p*<0.001] and unrelated conditions [*F*(1, 144)=35.82, *p*<0.001].

In the L1 group, the reaction times (mean±*SD*) for the related, unrelated, and perceptual conditions were 877±163ms, 916±187ms, and 665±170ms. In the L2 group, the reaction times (mean±SD) for the related, unrelated, and perceptual conditions were 1,223±202ms, 1,418±285ms, and 787±245ms. A 2 group (L1, L2)×3 condition (related, unrelated, perceptual) ANOVA on reaction times was performed. The results showed a main effect of group [*F*(1, 48)=37.71, *p*<0.001], a main effect of condition [*F*(2, 96)=170.74, *p*<0.001], and an interaction between group and condition [*F*(2, 96)=29.70, *p*<0.001]. The further simple main effect analyses revealed that the L2 group had generally slower responses in the perceptual [*F*(1, 144)=4.18, *p*=0.044], related [*F*(1, 144)=33.26, *p*<0.001], and unrelated conditions [*F*(1, 144)=70.22, *p*<0.001] as compared to the L1 group.

### Whole-Brain Results

The whole-brain analysis for the contrast between related pairs and perceptual control pairs within the L1 group and L2 group is shown in Table 1. In the L1 group, compared to perceptual controls, semantic-related pairs produced greater activation in the left hemisphere, including the IFG, SFG, and STG, as well as bilateral caudates. In the L2 group, the same contrast elicited greater activation in the left IFG, left medial frontal gyrus, left fusiform gyrus, and right MOG. In addition, greater activation was found in the subcortical regions in the L2 group.

**Table 1:** Brain regions for the related versus perceptual contrast in the whole-brain analyses.

Regions	*H*	BA	Voxels	Value of *Z*	MNI coordinates		
					*x*	*y*	*z*
**L1 group**							
**Inferior frontal gyrus**	**L**	**45**	**1,506**	**6.16**	**−54**	**23**	**17**
Inferior frontal gyrus	L	47		5.51	−30	29	−1
Precentral gyrus	L	6		4.08	−42	2	45
**Superior frontal gyrus**	**L**	**6**	**534**	**4.56**	**−6**	**14**	**59**
Superior frontal gyrus	L	8		4.49	−6	23	55
Frontal pole	L	9		4.56	−6	53	45
**Superior temporal gyrus**	**L**	**22**	**220**	**4.64**	**−57**	**−43**	**3**
Middle temporal gyrus	L	21		4.58	−54	−37	−1
**Caudate**	**L**	**–**	**253**	**4.68**	**−9**	**11**	**10**
**Caudate**	**R**	**–**	**168**	**4.65**	**12**	**11**	**−1**
**L2 group**							
**Inferior frontal gyrus**	**L**	**45**	**1,108**	**5.68**	**−54**	**29**	**20**
Inferior frontal gyrus	L	44		5.44	−45	11	24
Inferior frontal gyrus	L	47		5.08	−36	32	−15
Precentral gyrus	L	6		3.72	−54	−1	48
**Medial frontal gyrus**	**L**	**8**	**339**	**4.25**	**−3**	**17**	**52**
Anterior cingulate cortex	R	32		3.63	9	26	34
**Fusiform gyrus**	**L**	**37**	**598**	**4.61**	**−42**	**−46**	**−15**
Middle occipital gyrus	L	19		4.01	−36	−94	13
**Middle occipital gyrus**	**R**	**37**	**161**	**4.37**	**42**	**−88**	**13**
**Pallidum**	**R**	**–**	**709**	**4.56**	**9**	**−1**	**−5**
Caudate	R	**–**		4.21	12	14	−1
Parahippocampal gyrus	R	**–**		3.34	9	−7	−19
Inferior frontal gyrus	R	**47**		3.91	27	26	−8
Midbrain	L	**–**		4.49	−3	−19	−22
Putamen	L	**–**		3.57	−15	5	−8
Parahippocampal gyrus	L	**–**		3.37	−9	−4	−19
Thalamus	I	**–**		3.19	0	−7	3
**L2 group>L1 group**							
**Fusiform gyrus**	**L**	**37**	**537**	**3.90**	**−42**	**−49**	**−15**
Middle occipital gyrus	L	19		3.42	−33	−88	3
Inferior occipital gyrus	L	19		3.15	−39	−79	−8
**Middle occipital gyrus**	**R**	**19**	**306**	**3.79**	**30**	**−70**	**34**
Inferior temporal gyrus	R	19		3.29	45	−67	−12
Superior parietal lobule	R	7		3.26	30	−61	48
**L1 group>L2 group**							
**Insula**	**L**	**–**	**721**	**4.22**	**−33**	**−16**	**10**
Temporal pole	L	**38**		3.00	−51	8	−15
Middle/Superior temporal gyrus	L	**21**		3.51	−66	−22	−1
**Supramarginal gyrus**	**L**	**40**	**111**	**3.14**	**−51**	**−49**	**24**
Superior temporal gyrus	L	41		3.06	−48	−46	20
**Insula**	**R**	**–**	**809**	**4.14**	**33**	**−13**	**6**
Putamen	R	**–**		4.10	30	−7	−1
Temporal pole	R	**38**		3.61	36	8	−22
Middle temporal gyrus	R	**21**		2.99	63	−16	−8
Superior temporal gyrus	R	**22**		3.24	66	−13	3

*All the reported areas were set to a voxel-wise threshold of p=0.005 and 100 cluster extent to achieve the FWE-corrected threshold of p<0.05 at the cluster level. H, Hemisphere; L, Left; R, Right; I, Interhemispheric; BA, Brodmann area. Bold areas for main peaks*.

**Figure 1:**
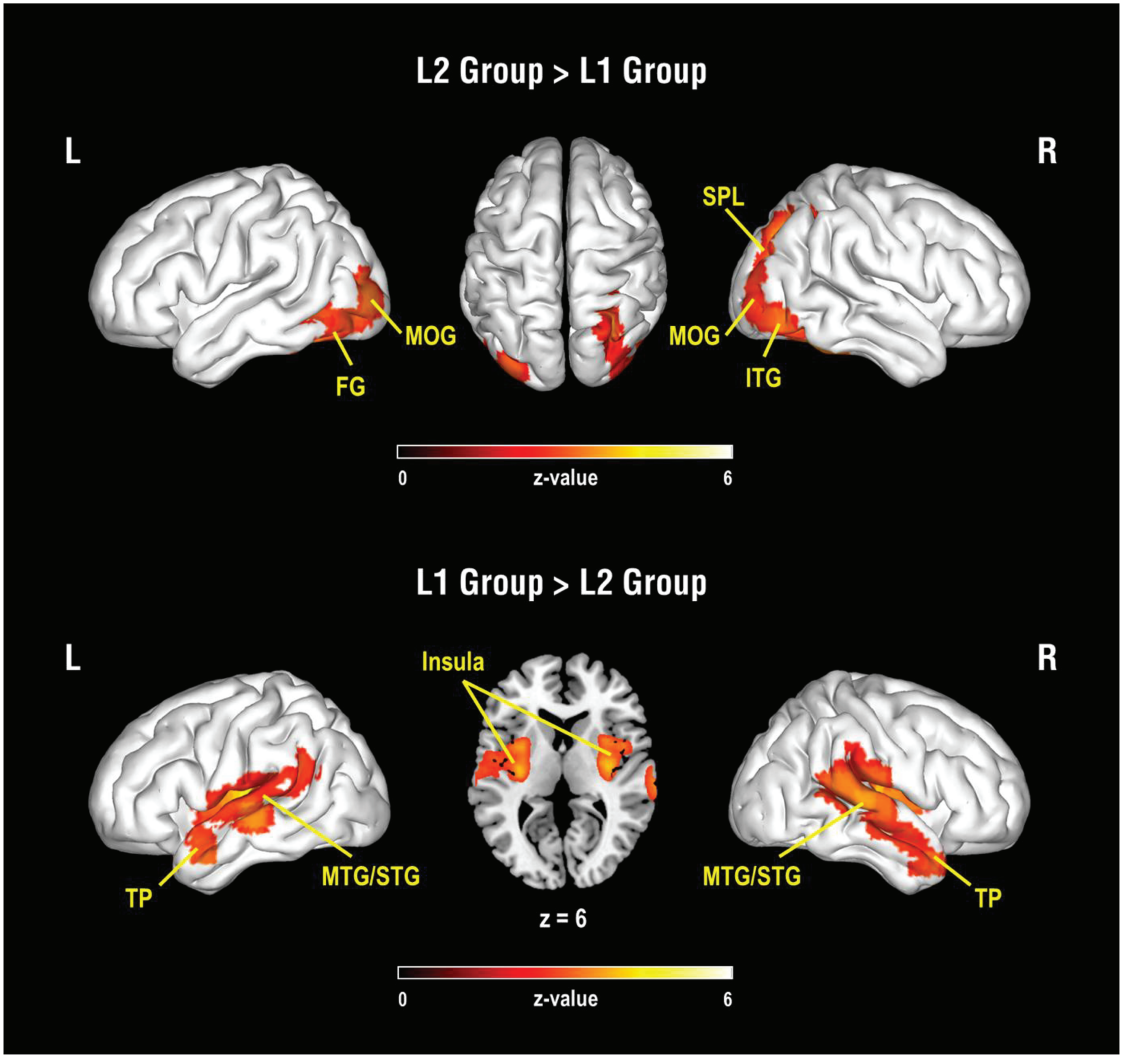
Results of group comparisons for the contrast of semantic-related condition vs. perceptual control condition. The upper panel is the activated brain areas for the comparison of the L2 group vs. L1 group, and the lower panel is the activated brain areas for the comparison of the L1 group vs. L2 group. L, Left hemisphere; R, Right hemisphere; FG, fusiform gyrus; MOG, middle occipital gyrus; SPL, superior parietal lobule; ITG, inferior temporal gyrus; TP, temporal pole; MTG, middle temporal gyrus; STG, superior temporal gyrus.

The results of the between-group analysis are shown in Table 1 and Figure 1. Compared to the L1 group, the L2 group showed greater activation near the occipital and parietal regions, including the left fusiform gyrus, bilateral MOG, right inferior temporal gyrus (ITG), and the right SPL in related pairs relative to perceptual control pairs. Also, compared to the L1 group, lower activation was found in the bilateral insulas and bilateral temporal regions, including the temporal pole, MTG, STG, and the left supramarginal gyrus in the L2 group.

### Correlation Results

**Figure 2:**
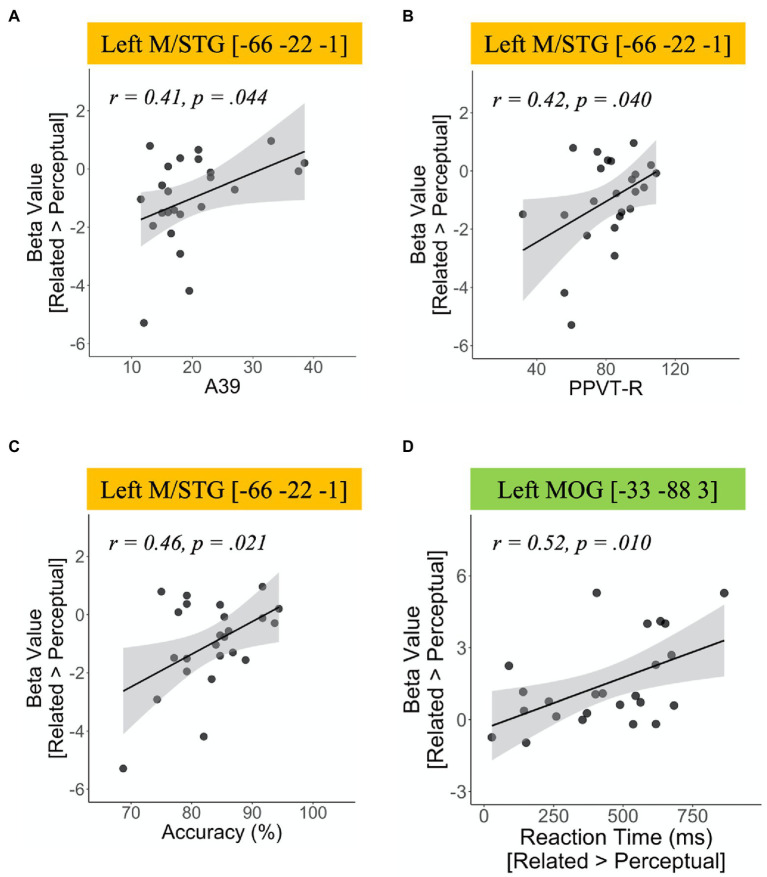
Correlation results of brain activation for the contrast of (semantic-related condition vs. perceptual control condition) in the L2 group. **(A)** Correlation between activation in the left middle temporal gyrus/superior temporal gyrus (M/STG) and Peabody Picture Vocabulary Test-Revised scores, **(B)** correlation between activation in the left M/STG and A39 scores, **(C)** correlation between activation in the left M/STG and accuracy, and **(D)** correlation between activation in the left middle occipital gyrus and reaction time.

To investigate the role of individual differences in task performance and vocabulary knowledge during the semantic processing in the L2 group, we extracted the peak activation in ROIs from the group comparison and then correlated the values with the behavioral accuracy in the fMRI task and the scores in the two vocabulary tests. The correlation results are shown in Figure 2. Greater activation in the left M/STG was positively correlated with behavioral accuracy (*r*=0.46, *p*=0.021) and the scores in both vocabulary tests (A39 test: *r*=0.41, *p*=0.044; PPVT-R test: *r*=0.42, *p*=0.04). In addition, we found a positive correlation between response time and the activation in the left MOG (*r*=0.52, *p*=0.010).

### Connectivity Results

**Figure 3:**
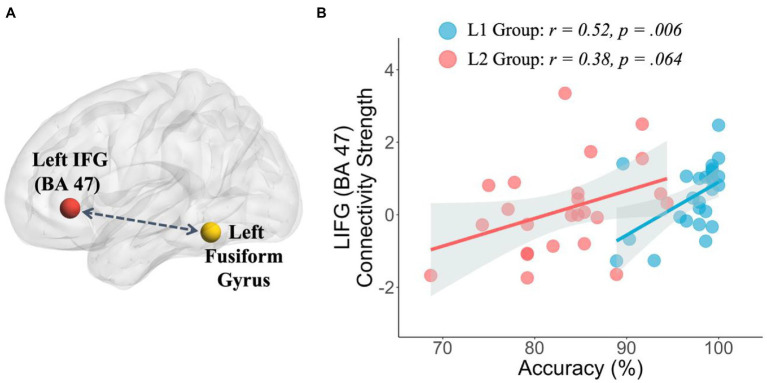
Results of region of interest-based generalized psychophysiological interaction connectivity analysis. **(A)** Visualization of the seed region (yellow sphere) and the target region (red sphere) and **(B)** correlation results between behavioral accuracy in the semantic judgment task and connectivity strength for the L1 group and L2 group.

We used the gPPI analysis to investigate the functional connectivity between the left fusiform gyrus and left ventral IFG (BA 47) during semantic processing in both L1 and L2 groups (see Figure 3). Compared to the L1 group, the L2 group showed a relatively reduced connectivity strength between brain regions; however, there was no significant difference between the two groups (*t*(48)=1.16, *p*=0.254). We further correlated the connectivity strengths with participants’ performance in the semantic judgment task for the L1 group and L2 group, respectively. Our results revealed that the L1 group showed a positive correlation between their task performance and connectivity strengths (*r*=0.52, *p*=0.006), whereas the L2 group showed a similar trend but with a marginal significance (*r*=0.38, *p*=0.064).

## Discussion

The present study investigated long-term learning effects on the ventral pathway with semantic processing, a prominent feature of Chinese words. We found the group differences in the neural mechanisms of semantic processing between native Chinese speakers (L1 group) and proficient adult learners of Mandarin Chinese (L2 group). Compared to the L1 group, the L2 group showed greater activation in the bilateral areas adjacent to the fusiform gyrus and MOG in the semantic-related condition relative to the perceptual control condition. In contrast, the L2 group showed reduced activation in the temporal regions on both hemispheres as compared to the L1 group. In addition, we found individual differences in brain activity along the ventral pathway in the L2 group. By correlating the brain activation with the participants’ performances in the vocabulary tests and the semantic judgment task, increased activation in the left posterior M/STG was positively associated with the L2 participants’ proficiencies in semantic knowledge. Furthermore, we found the group differences in brain interaction such as functional connectivity along the ventral pathway between groups. The positive correlation between participants’ accuracy in semantic judgments and the functional connectivity in the ventral pathway (left fusiform-left IFG) was robust in the L1 group but weaker in the L2 group.

Consistent with previous findings in reading Chinese characters, our results demonstrated that L2 participants recruited more areas in the occipital lobes, including the left fusiform gyrus, bilateral MOG, and right ITG, as well as the right SPL. Previous studies suggested that reading Chinese characters requires more involvement of the bilateral fusiform gyri and MOG to fulfill the higher demand of orthographic processing (Bolger et al., 2005; Nelson et al., 2009; Mei et al., 2015). The right SPL and right ITG has also been linked to visual-orthographic analysis during language processing. Cao et al. (2010) found that Mandarin Chinese speakers elicited greater activation in the right SPL and right ITG in the spelling task than the rhyming task, suggesting that these regions are associated with the visuo-orthographic analysis of Chinese characters. One of our correlation results also showed a positive trend between greater activation in the left MOG and slower reaction time in the semantic-related condition relative to the perceptual control condition. Previous studies have shown that children with dyslexia generally have reduced activation in the MOG regions, indicating their deficit in recruiting the MOG for visual processing (Boros et al., 2016). Cao et al. (2018) also found that compared to the control groups, children with developmental dyslexia had weaker functional connectivity between the left MOG and left IFG in the lexical task, suggesting that they have impairments in dealing with the orthography-phonology relations of Chinese characters. Thus, the relation between slower reaction time and higher activation in the left MOG in the L2 group could be a trade-off for visuospatial analysis of Chinese characters. That is, L2 participants who engaged greater neural resources in processing orthographic information of Chinese characters might spend more time during the semantic judgment.

Another major finding in the present study is the individual proficiency of semantic knowledge modulated activation in the posterior temporal region in the L2 participants. As a group, L2 participants showed reduced activation in the bilateral temporal lobes compared to the L1 group. However, the correlation results exhibited that L2 participants’ scores in the vocabulary tests and the performances in the semantic judgment task were positively associated with activation in the left posterior M/STG (BA 21). Several language models suggested that the left posterior MTG (BA 21) and the adjacent areas are critical regions for the storage of lexical-semantic representations in our brain (Hickok and Poeppel, 2007; Lau et al., 2008; Binder et al., 2009). Wei et al. (2012) using resting-state fMRI found that the low-frequency fluctuations of the BOLD signal in the left posterior MTG were correlated with native speakers’ efficiency of semantic processing. Developmental studies also demonstrated that greater activation in the left posterior MTG was associated with children’s ages (Chou et al., 2006a,b; Lee et al., 2015, 2016), indicating that the greater elaboration of semantic representations with increasing age during language development. Our study corroborated the functional role of the posterior M/STG (BA 21) by correlating brain activation with L2 participants’ proficiency in semantic knowledge, assessed by the vocabulary tests and the performance in the semantic judgment task.

The gPPI connectivity analysis in the current study revealed that functional connectivity between the left fusiform and left ventral IFG (BA 47) was positively correlated with participants’ accuracy in the semantic judgment task in the L1 group. Previous studies using the diffusion tensor imaging technique have demonstrated that the left inferior fronto-occipital fasciculus, a ventral white matter bundle that connects the frontal and occipital regions, is associated with the performance in the lexical/semantic-related tasks in both healthy adult participants (Nugiel et al., 2016) and brain-damaged patients (Han et al., 2013). Our functional connectivity results in the L1 group provided convergence evidence that the ventral pathway on the left hemisphere plays an important role in Lexico-semantic processing. Moreover, the individual semantic abilities would modulate the connectivity strengths in this ventral pathway.

By contrast, the L2 group showed a similar but weaker trend between task performance and functional connectivity in the ventral pathway (left fusiform-left IFG). In Wang et al.’s (2019) study, they found that functional connectivity between the left ventral occipitotemporal cortex and left IFG (BA 47) was associated with semantic processing during Chinese word recognition, suggesting that, for Mandarin Chinese native speakers, this ventral pathway is related to orthographic-semantic mapping during reading. Fan et al. (2020) also found that, compared to adults, children had weaker functional and structural connectivity in the left ventral language pathway, indicating that this left ventral language pathway matures with increasing age during children’s development. The L2 participants in the current study were all Indo-European native speakers who started learning Mandarin Chinese in adulthood. Thus, taking the perspective from language development and considering the fact that adults have generally reduced neural plasticity, late learners of Mandarin Chinese might need more time and L2-related experience to develop and to strengthen the connectivity in this ventral pathway for orthographic-semantic processing when reading Mandarin Chinese.

The findings and interpretations of the present study should be considered in light of its limitations. For example, these two groups might differ in several aspects, such as age of acquisition and learning methods. Thus, it is hard to disentangle the effect of these factors in the present study. An ongoing project examines the learning effects in a longitudinal approach that participants in the L2 group receive a similar curriculum to learn Chinese. Comparing before versus after learning may provide a better way to understand the long-term effects on the ventral pathway in the L2 group. Also, L2 participants’ native language experience from different countries might limit the generalizability of our findings. During the participant recruitment process, we excluded L2 participants who were native speakers of Japanese, Korean, or other Asian languages due to the similarities between their native languages and Mandarin Chinese. In addition, we only recruited the L2 participants who were native speakers of the Indo-European languages to try to minimize the influence from their native languages. Future research should find a better-match group, such as Mandarin Chinese learners who have the same native language, to control the effects of different language backgrounds.

In sum, the present study demonstrated that proficient L2 adult learners of Mandarin Chinese recruited the bilateral brain regions in the occipital lobe, such as the fusiform gyrus, MOG, and right SPL for processing visuospatial information of Chinese characters in support of semantic processing. In addition, by correlating with L2 participants’ performances in vocabulary tests and the semantic judgment task, our results corroborated the functional role of the left posterior MTG in the storage of semantic representations. Moreover, functional connectivity between the left fusiform gyrus and left ventral IFG (BA 47) was modulated by performance in the semantic judgment task in the L1 native speakers. However, L2 adult learners only exhibited a weaker trend of this modulation effect during semantic processing in Mandarin Chinese.

## Data Availability Statement

The original contributions presented in the study are included in the article/supplementary material, further inquiries can be directed to the corresponding author.

## Ethics Statement

The studies involving human participants were reviewed and approved by the Research Ethics Committee at the National Taiwan University. The patients/participants provided their written informed consent to participate in this study.

## Author Contributions

All authors took part in data collection. C-HL analyzed the data and wrote the manuscript. S-KH, C-LL, LS, T-HL, C-RL, and I-NT provided critical thoughts and reviewed the manuscript. T-LC designed the study and provided guidance for data analysis and manuscript writing. All authors contributed to the article and approved the submitted version.

## Funding

This work was supported by a grant from the Ministry of Science and Technology of Taiwan (MOST 105-2420-H-002-007-MY2) and a grant from the Higher Education Sprout Project (NTU-110L104038).

## Conflict of Interest

The authors declare that the research was conducted in the absence of any commercial or financial relationships that could be construed as a potential conflict of interest.

## Publisher’s Note

All claims expressed in this article are solely those of the authors and do not necessarily represent those of their affiliated organizations, or those of the publisher, the editors and the reviewers. Any product that may be evaluated in this article, or claim that may be made by its manufacturer, is not guaranteed or endorsed by the publisher.

## Footnotes

^1^The native languages of participants in the L2 group include English (6), Spanish (6), French (3), Russian (2), Dutch (1), Swedish/Dutch (1), Lithuanian (1), Turkish (1), Danish (1), Hungarian (1), and Italian (1). The number in the parenthesis indicates the number of native speakers of a certain language.

^2^ https://tocfl.edu.tw/index.php
